# Shifting Mountain Tree Line Increases Soil Organic Carbon Stability Regardless of Land Use

**DOI:** 10.3390/plants13091193

**Published:** 2024-04-25

**Authors:** Sofia Sushko, Kristina Ivashchenko, Alexandra Komarova, Anna Yudina, Victoria Makhantseva, Ekaterina Elsukova, Sergey Blagodatsky

**Affiliations:** 1 Institute of Physicochemical and Biological Problems in Soil Science, Russian Academy of Sciences, Pushchino 1422902, Russia; ivashchenko.kv@gmail.com (K.I.); alexandra_seleznyova@mail.ru (A.K.); victoriamakhantceva@gmail.com (V.M.); 2 V.V. Dokuchaev Soil Science Institute, Moscow 119017, Russia; yudina_av@esoil.ru; 3 Department of Geoecology, Institute of Earth Sciences, Saint Petersburg State University, Saint-Petersburg 199034, Russia; elsukovaeu@mail.ru; 4 Terrestrial Ecology Group, Institute of Zoology, University of Cologne, 50674 Cologne, Germany; sblag11@gmail.com

**Keywords:** particulate organic matter, mineral-associated organic matter, aboveground grass biomass, forest litter, ^13^C-NMR spectroscopy, aromaticity index, C/N ratio

## Abstract

Climate and land use changes are causing trees line to shift up into mountain meadows. The effect of this vegetation change on the partitioning of soil carbon (C) between the labile particulate organic matter (POM–C) and stable mineral-associated organic matter (MAOM–C) pools is poorly understood. Therefore, we assessed these C pools in a 10 cm topsoil layer along forest–meadow ecotones with different land uses (reserve and pasture) in the Northwest Caucasus of Russia using the size fractionation technique (POM 0.053–2.00 mm, MAOM < 0.053 mm). Potential drivers included the amount of C input from aboveground grass biomass (AGB) and forest litter (litter quantity) and their C/N ratios, aromatic compound content (litter quality), and soil texture. For both land uses, the POM–C pool showed no clear patterns of change along forest–meadow ecotones, while the MAOM–C pool increased steadily from meadow to forest. Regardless of land use, the POM–C/MAOM–C ratio decreased threefold from meadow to forest in line with decreasing grass AGB (R^2^ = 0.75 and 0.29 for reserve and pasture) and increasing clay content (R^2^ = 0.63 and 0.36 for reserve and pasture). In pastures, an additional negative relationship was found with respect to plant litter aromaticity (R^2^ = 0.48). Therefore, shifting the mountain tree line in temperate climates could have a positive effect on conserving soil C stocks by increasing the proportion of stable C pools.

## 1. Introduction

Changes in climate and land uses are the main triggers of vegetation cover shifts [1]. In this respect, the hotspot is mountain tree lines, expanding into meadows with a rate of up to 0.4–3.6 m per year in different regions [2,3]. These vegetation shifts are associated with qualitative and quantitative changes in plant litter input in soils, thus potentially altering soil organic carbon (C) stocks. Previous studies have shown that the topsoil C stocks (0–10/0–30 cm layers) of mountain meadows are more vulnerable to forest expansion [4] than those in the whole soil profile [5,6]. However, to accurately predict the dynamics of soil C stocks generated by tree line shifting, it is important to understand the relative changes in their constituent pools, which are contrasting in availability for microbial decomposition and mean residence time. Among these C pools, particulate organic matter (POM–C) and mineral-associated organic matter (MAOM–C) are currently being increasingly considered under climate and land use changes [7,8]. POM–C is mainly formed from the fragmentation of plant litter that is in free or occluded soil aggregates with a short residence time (<10 years-decades). In contrast, MAOM–C encompasses single organic molecules derived from microorganisms and plants that are strongly associated with minerals and have a long residence time (from decades to centuries) [8]. Thus, POM–C is attributed to the relative labile soil C pool, and MAOM–C is credited to the stable and long-living one.

Several researchers have assessed POM–C and MAOM–C dynamics under mountain tree line shifts [9,10]. They showed that forest expansion into meadows reduced POM–C pools, whereas MAOM–C remained either unchanged [9] or slightly decreased [10]. Nevertheless, the contribution of plant litter quantity and quality to soil C accumulation in POM and MAOM under different land uses was not studied in these works. Tree expansion into meadows is known to primarily decrease grass density and productivity [11,12], which can reduce the contribution of this plant litter source to soil C pools. Recalcitrant forest litter can also promote net C gains in both POM–C and MAOM–C [13]. However, the chemical composition of POM–C is closer to that of the initial plant material than MAOM–C [10,14], suggesting that litter characteristics make a greater contribution to its formation. The effect of plant litter quality on soil C pool dynamics was found to be specific to soil texture [15]. It is known that the abundance of fine mineral particles directly determines the MAOM–C pool [7,16]. Because the translocation of fine particles with runoff can occur along mountain slopes [17], this may additionally contribute to changes in POM–C versus MAOM–C under conditions of tree line shifting. In addition, grazing on mountain pastures can further increase the redistribution of fine particles along a slope by increasing soil loss through erosion. Therefore, we hypothesized that (i) changes in POM–C along mountain forest–meadow ecotones are mainly driven by the quantity and quality of plant litter entering the soil and that (ii) changes in MAOM–C, in contrast, are more driven by soil texture, which is related to both topography (location on a slope) and grazing. To test these hypotheses, our study focused on examining the changes in the topsoil POM–C/MAOM–C ratio along the forest–meadow ecotones of reserve and pasture mountain slopes and assessing their relationship with C input from aboveground grass biomass and forest litter, their C/N ratios and aromatic compound content, and silt and clay content.

## 2. Results

### 2.1. Plant Aboveground Litter and Soil Texture

**Figure 1:**
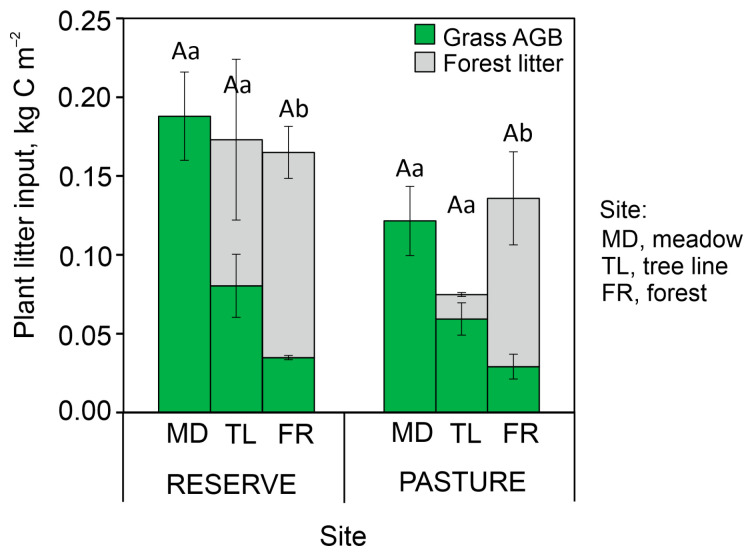
Plant litter input from grass aboveground biomass (AGB) and forest litter along forest–meadow ecotones with different land uses. Mean ± standard error for *n* = 3; different lowercase letters show significant differences for grass AGB, and capital letters show these differences for total plant litter input (grass AGB + forest litter) for each land use.

The C input with grass aboveground biomass (AGB) decreased 4–5 times from meadows to forests for both land uses (Figure 1). Simultaneously, the C storage in total plant litter (grass AGB + forest litter) did not differ significantly along the forest–meadow ecotones. For both land uses, the C content of aromatic compounds in the total plant litter gradually increased from the meadows to the forests (Table 1) due to the contribution of forest litter (Table A1). At the same time, the C/N ratio of total plant litter showed a consistent opposite trend despite its insignificance: a decrease from the meadows to the forests.

**Figure 2:**
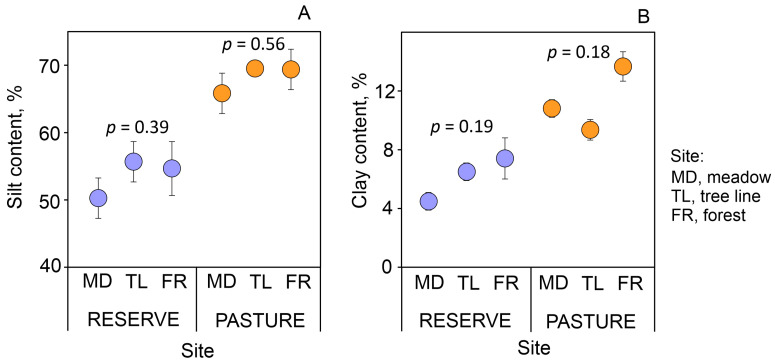
Silt (0.002–0.05 mm) (**A**) and clay (<0.002 mm) (**B**) content in topsoil (0–10 cm) along forest–meadow ecotones with different land uses. Mean ± standard error for *n* = 3; *p*-value for Kruskal–Wallis test.

For both land uses, the content of silt and clay for the forests was slightly higher than that for the meadows, but the differences were not significant (Figure 2). The soil texture of all the studied sites was classified as silt loam, but the average content of silt and clay was higher in the pasture than in the reserve (*p* < 0.001 for Welch’s *t*-test).

### 2.2. Topsoil C Stock Distribution between POM–C and MAOM–C Pools

**Figure 3:**
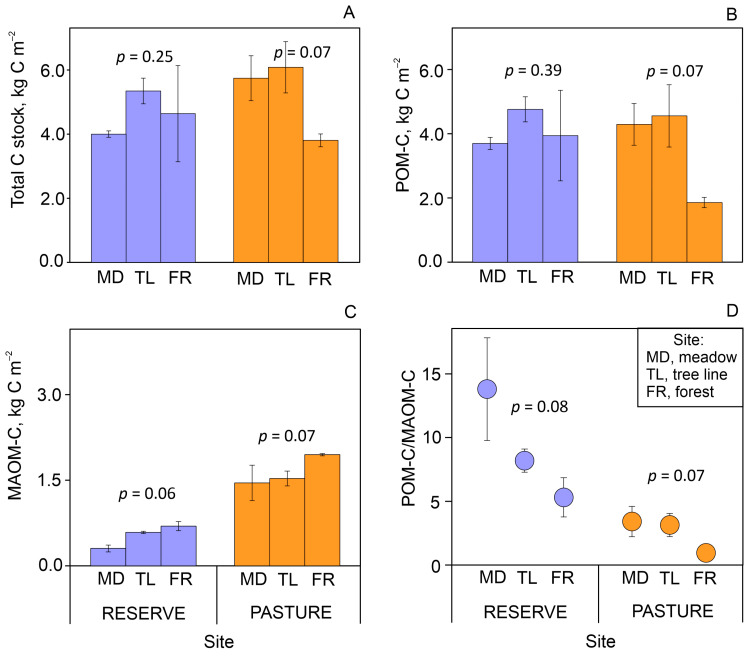
Total C stock in 10 cm topsoil layer (**A**), its distribution in particulate organic matter (POM–C) (**B**) and mineral-associated organic matter (MAOM–C) pools (**C**), and POM–C/MAOM–C ratios (**D**) along forest–meadow ecotones with different land uses. Mean ± standard error for *n* = 3; *p*-value for Kruskal–Wallis test.

The total C stocks in the 10 cm topsoil layer did not change significantly along the forest–meadow ecotones and amounted to 4.0–5.3 and 3.8–6.1 kg C m^−2^ for the reserve and pasture sites, respectively (Figure 3A). Labile POM–C accounted for 82–92% and 49–74% of the total C stocks in the reserve and pasture, respectively (Table A2). Insignificant changes in the POM–C pool along the tree line gradient were synchronous with the total C stock (Figure 3B). Stable MAOM–C showed a clear increasing pattern from meadow to forest for both land uses (Figure 3C). As a result, the POM–C/MAOM–C ratio tended to decrease threefold from meadow to forest in the reserve and pasture sites (Figure 3D). This suggests that the stability of topsoil C stocks increases with mountain tree line shifting, regardless of land use.

**Figure 4:**
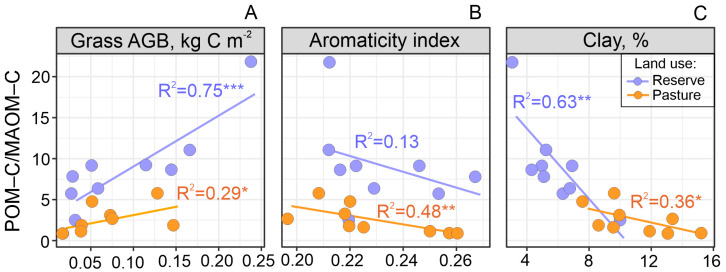
Relationship of topsoil POM–C/MAOM–C ratio (0–10 cm) with grass aboveground biomass (AGB) (**A**), plant aromaticity index (**B**), and clay content (**C**) along forest–meadow ecotones with different land uses (* *p* ≤ 0.1; ** *p* ≤ 0.05; *** *p* ≤ 0.01).

Correlation analysis showed that POM–C/MAOM–C ratios were significantly associated with grass AGB (*r* = 0.86), plant C/N ratios (*r* = 0.69), and clay content (*r* = −0.76) for the reserve sites and with plant aromaticity index (*r* = −0.68) for the pasture sites (Table A3). As the plant C/N ratios on the reserves were strongly correlated with grass AGB (*r* = 0.87), they were not included in further regression analyses. For both land uses, changes in the POM–C/MAOM–C ratio along forest–meadow ecotones were positively associated with the amount of grass AGB (Figure 4A) and negatively associated with the clay content (Figure 4C). These relationships were stronger for the reserves (R^2^ = 0.75 and 0.63) than for the pastures (R^2^ = 0.29 and 0.36). At the same time, a negative relationship between POM–C/MAOM–C and the plant aromaticity index (R^2^ = 0.48) was revealed only in the pastures (Figure 4B). Hence, for both land uses, mountain tree line shifting decreased POM–C in line with a decreasing grass litter input and, conversely, increased MAOM–C in line with an increasing soil clay content and aromaticity of total plant litter (pasture only). This partially confirms our hypotheses that the labile POM–C pool along forest–meadow ecotones is mostly determined by plant litter characteristics and that the stable MAOM–C pool is determined by soil texture.

## 3. Discussion

Changes in the topsoil POM–C/MAOM–C ratio from the mountain meadow to the forest transect were positively correlated with the amount of grass AGB (Table A3). However, using total litter (forest and grass) in the analysis weakened this relationship. It follows that the annual C input from aboveground grass litter is a greater contributor to POM–C formation than that from forest litter. This can be associated with their different types of biochemical recalcitrance with respect to enzymatic degradation, which generally increases with the concentration of complex aromatic polymers (i.e., lignins and tannins) in plant litter [18]. In the warm conditions of lowlands, the decay of deciduous forest litter, particularly its lignified components such as sticks and bark, can continue for several years [19], while 95% of the grass litter (leaves and stems) can be microbially utilized in 1 year [20]. In mountain conditions, the short and cool growing season limits the rapid microbial utilization of the grass litter, extending up to 2–5 years [21]. Nevertheless, it can be assumed that the real amount of aboveground litter annually involved in the soil C pools was higher in meadows than in forests for the studied mountain slopes (Figure 1).

In high mountain meadows, the role of abiotic factors in the physical fragmentation of abundant grass litter is increasing, such as with respect to freeze–thaw events and intensive solar radiation [21]. The latter, i.e., photodegradation, mainly contributes to the breakdown of lignins [18], which is quite high in some alpine grasses [22]. Thus, slow biotic degradation together with the abiotic fragmentation of abundant meadow litter forms a POM-dominated topsoil (0–5/0–10 cm), as shown in previous studies [23,24] and confirmed by our results (Figure 3B, Table A2). Hence, the decreasing amounts of aboveground grass litter as forests expand would be expected to reduce the POM–C fraction in topsoil C stocks. That said, a higher content of fine-sized particles (clay and silt) in forests contributes to a higher MAOM–C fraction. Such changes in soil texture are indirectly associated with a decrease in grass cover, the density of which largely controls soil erosion intensity on the slopes [25]. In addition, rain splash erosion of forest soils can increase due to the increased size and kinetic energy of raindrops passing through the tree canopy [26]. Soil erosion exposes mineral materials, increasing their further weathering and hence clay formation [27]. In our study, MAOM–C correlated more closely with clay-sized particles (<0.002 mm) than with silt-sized ones (0.002–0.05 mm) (Table A3). This can be related to the larger surface area of clay particles, and therefore their greater saturation capacity with respect to organic matter, or to the differing mineralogy of clay and silt particles [28]. Furthermore, the more recalcitrant and chemically diverse forest litter than in the polysaccharide-dominated meadow AGB (Table A1) leads to a greater functional diversity of the soil microbial community [29]. This, in turn, provides a greater variety of organic molecules released during the forest litter decomposition. Considering the selective sorption of various organic molecules by minerals [30], the potential for their stabilization on clay-sized particles increases with their diversity. Thus, in addition to increasing clay content, changes in plant litter quality can also contribute to MAOM–C dynamics under conditions of mountain tree line shifting. In general, our results have shown that shifting a mountain tree line has a positive effect on conserving soil C stocks by increasing the proportion of stable C pools, regardless of land use.

## 4. Materials and Methods

### 4.1. Study Area and Sampling Design

**Figure 5:**
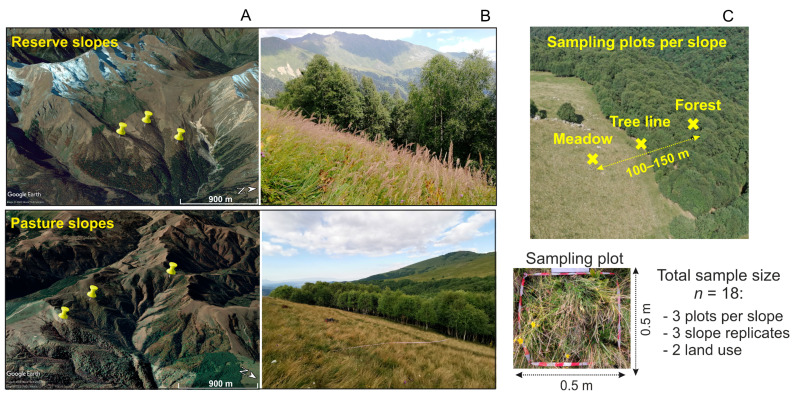
Scheme of location (**A**) and general view (**B**) of the studied reserve and pasture slopes in the Northwest Caucasus of Russia; sampling design (**C**).

**Figure A1:**
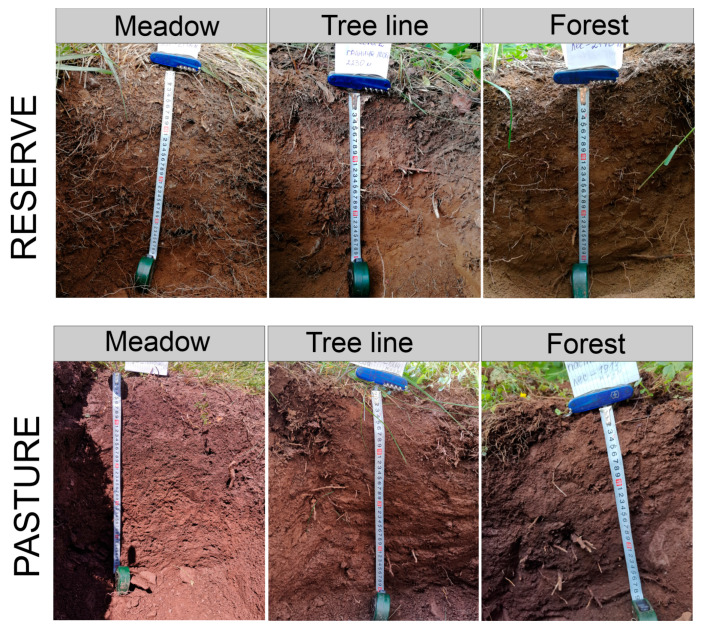
Soil profiles along studied forest–meadow ecotones with different land uses.

Six forest–meadow ecotones were chosen in the Northwest Caucasus of Russia: 3 on reserve slopes at 2130–2230 m a.s.l. (43°43′ N; 40°43′ E) and 3 others on pasture slopes at 1880–1940 m a.s.l. (43°43′ N; 41°12′ E) (Figure 5). The reserve slopes were located in the Caucasian State Nature Biosphere Reserve [31]; the pasture slopes were used for long-term (>100 years) grazing by cattle, horses, and sheep. All studied slopes had northeastern exposure, with a steepness of 25–30°. The climate of the study area is moderately continental, with average annual air temperatures of 5.1–8.5 °C (according to our measurements taken at the study sites for 2020–2021 using DS1922L-F5 iButton^®^ logger, Maxim Integrated, San Jose, CA, USA) and annual precipitation of 1850 mm (according to the closest meteorological station located at 2006 m a.s.l.; 43°15′ N; 41°50′ E). Soils were predominantly Haplic (Humic) Cambisols (Figure A1) according to FAO soil classification [32]. Parent materials were non-alkaline rocks, that is, andalusite–mica schists, amphibolite depositions, and mudstones [33]. The topsoil (0–10 cm) of the studied slopes was carbon-rich (with a C content ranging from 7.7 to 14.2%) and very strongly acidic (with a pH from 4.6 to 5.0), as shown in our previous study [34].

For each slope, a 100–150 m transect crossing meadow, tree line, and forest vegetation types was selected. Along each transect, 3 plots of 0.5 × 0.5 m were equally spaced; that is, there was one plot per vegetation type (Figure 5C). The forests subjected to both land uses consisted of deciduous trees predominantly represented by *Betula* sp. The ages of the reserve trees were 45–60 and 116–122 at tree line and forest sites, respectively. Pasture trees were younger, reaching 18–22 and 60–75 years of age at tree line and forest sites, respectively. For both land uses, the grass cover of the meadows and tree lines was predominantly represented by graminoids (*Calamagrostis arundinacea* (L.) Roth, *Nardus stricta* L., and *Agrostis capillaris* L.), and that of the forests was mainly represented by forbs (e.g., *Aconitum orientale* Miller, *Prenantes abietina* (Boiss. et Balansa) Kirp., *Alchemilla vulgaris* L., and *Oxalis acetosella* L.) (Table A4). To assess the quantity and quality of plant residues entering the soil, the AGB of the grass cover (grass AGB) and forest litter were collected for each plot in mid-August 2020. After that, a soil sample from the upper 0–10 cm layer was taken per plot with a shovel. Additionally, 2 samples (0–5 and 5–10 cm layers) were taken using a steel cylinder with a volume of 88 cm^3^ to measure the soil bulk density. The soil samples were air-dried at 22 °C, passed through a 2 mm sieve to exclude roots and stones, and subsequently used for physicochemical analyses.

### 4.2. Plant and Soil Samples Analysis

Grass AGB and forest litter were dried at 50 °C and weighed to calculate their dry mass per unit area. Thereafter, the plant material was ground into fine powder in a mill and used for chemical analysis. Carbon (C) and nitrogen (N) content in the plant samples was measured using an automated “vario EL cube” analyzer (Elementar Analysensysteme GmbH, Langenselbold, Germany), after which the C/N ratio was calculated. The C portion of aromatic compounds in plant organic matter was determined via solid-state CP/MAS ^13^C-NMR analysis using Bruker Avance III 400WB spectrometer (Bruker Corporation, Billerica, MA, USA) with a frequency of 100.53 MHz (Magnetic Resonance Research Centre, Saint Petersburg State University, Russia). The CP/MAS ^13^C-NMR spectra were processed by integrating the signal intensity in specific chemical shift ranges corresponding to the following C functional groups: alkyl (0–45 ppm), O-alkyl (0–110 ppm), and aromatic (110–165 ppm) [35]. The first two C groups are associated with the presence of alkanes, fatty acids, waxes, proteins, peptides, and polysaccharides, and the third C group is associated with the presence of tannins and lignins. The ratios of these organic groups, that is, aromatic/(alkyl + O-alkyl + aromatic), constitute the aromaticity index [36].

Soil bulk density was calculated as the mass of completely dried samples (105 °C, 8 h) per unit volume. The soil texture was determined in soil suspensions after their ultrasonic treatment (total energy 450 J mL^−1^) via laser diffraction analysis using a Microtrac S3500 Bluewave (Microtrac Inc., Montgomeryville, PA, USA) [37]. Texture data were analyzed according to the percentage of particle size classes: clay (<0.002 mm), silt (0.002–0.05 mm), and sand (0.05–2.0 mm). Soil texture was classified according to USDA guidelines [38]. Physical fractionation of soil organic matter into POM and MAOM was performed using wet sieving through a 53 µm sieve [39]. Preliminary soil samples were dispersed using 0.5% solution of sodium hexametaphosphate (30 mL per 10 g of soil) and suspension shaking with the addition of glass beads (to facilitate better destruction of aggregates) for 15 h at 180 rpm. Both fractions were dried at 55 °C for at least 48 h and weighted, and C content was determined in the same way as for the plant samples. The C stocks in the POM and MAOM fractions were calculated, taking into account soil bulk density. The sum of POM–C and MAOM–C was considered to be 100% of total soil C stock, neglecting the contribution of the water-soluble pool, which generally accounts for <1% [40].

### 4.3. Data Analysis

Significance of differences in the studied plant residue characteristics and soil variables among sites (3 independent groups; *n* = 3 per group) was examined using the non-parametric Kruskal–Wallis test and Dunn’s test. Significance of differences in soil texture between land uses (2 independent groups; *n* = 9 per group) was examined using Welch’s *t*-test. The relationships between POM–C/MAOM–C ratio and plant litter characteristics and soil texture for each land use (*n* = 9) were tested using Pearson correlation analysis and linear regression analysis.

## Figures and Tables

**Table 1:** Portions of aromatic C, aromaticity index, and C/N ratio in total plant litter (grass AGB + forest litter) along forest–meadow ecotones with different land uses (mean ± standard error for *n* = 3; *p*-value for Kruskal–Wallis test; different letters show significant differences for Dunn’s test at *p* ≤ 0.05).

Site	Reserve			Pasture		
	Aromatic C, %	Aromaticity	C/N	Aromatic C, %	Aromaticity	C/N
Meadow	18.6 ± 0.1 ^b^	0.22 ± 0.00	33 ± 2	18.3 ± 0.5 ^b^	0.21 ± 0.01 ^b^	29 ± 1
Tee line	20.2 ± 0.7 ^ab^	0.23 ± 0.01	23 ± 0	19.3 ± 0.2 ^ab^	0.22 ± 0.00 ^ab^	28 ± 2
Forest	21.7 ± 1.1 ^a^	0.25 ± 0.01	23 ± 0	22.3 ± 0.3 ^a^	0.26 ± 0.00 ^a^	26 ± 1
*p*-value	0.05	0.06	0.07	0.03	0.04	0.25

## Data Availability

Detailed data of this research can be obtained by contacting the corresponding author.

## Appendix A

**Table A1:** Portions (%) of C content in different functional groups (^13^C-NMR spectroscopy), aromaticity index, and C/N ratio in plant litter along forest–meadow ecotones with different land uses (mean ± SE for *n* = 3).

Site	Alkyl C (0–45 ppm)	O-Alkyl C (45–110 ppm)	Aromatic C (110–165 ppm)	Aromaticity	C/N
Reserve					
Grass AGB:					
Meadow	5.6 ± 0.3	62.3 ± 0.5	18.6 ± 0.1	0.22 ± 0.00	33 ± 2
Tree line	6.2 ± 0.1	61.2 ± 0.5	18.9 ± 0.2	0.22 ± 0.00	24 ± 1
Forest	10.4 ± 0.8	58.8 ± 1.0	16.7 ± 0.5	0.19 ± 0.01	22 ± 1
Forest litter	14.9 ± 1.0	49.8 ± 1.1	23.0 ± 1.1	0.26 ± 0.01	24 ± 1
Pasture					
Grass AGB:					
Meadow	6.9 ± 0.5	62.1 ± 1.0	18.3 ± 0.5	0.21 ± 0.01	29 ± 1
Tree line	7.8 ± 0.7	61.2 ± 0.9	18.1 ± 0.1	0.21 ± 0.00	28 ± 2
Forest	8.0 ± 0.3	63.1 ± 2.3	17.4 ± 0.5	0.20 ± 0.01	25 ± 3
Forest litter	14.1 ± 0.7	48.0 ± 1.7	23.8 ± 0.7	0.28 ± 0.01	27 ± 1

**Table A2:** Carbon content in particulate organic matter (POM–C) and mineral-associated organic matter (MAOM–C) in 10 cm topsoil layer along forest–grassland ecotones with different land uses (mean ± SE for *n* = 3).

Site	POM–C			MAOM–C		
	g kg^−1^	kg C m^−2^	% of Total C	g kg^−1^	kg C m^−2^	% of Total C
Reserve						
Meadow	148 ± 16	3.7 ± 0.2	92 ± 2	12 ± 3	0.3 ± 0.1	7 ± 2
Tree line	116 ± 12	4.8 ± 0.4	89 ± 1	14 ± 0	0.6 ± 0.0	11 ± 1
Forest	93 ± 26	3.9 ± 1.4	82 ± 5	18 ± 0	0.7 ± 0.1	18 ± 5
Pasture						
Meadow	81 ± 9	4.3 ± 0.7	74 ± 6	28 ± 7	1.5 ± 0.3	26 ± 6
Tree line	85 ± 12	4.6 ± 1.0	73 ± 6	30 ± 6	1.5 ± 0.1	27 ± 6
Forest	33 ± 2	1.9 ± 0.2	49 ± 2	34 ± 1	1.9 ± 0.0	51 ± 2

**Table A3:** Pearson’s correlation of POM-C/MAOM-C ratio with plant litter characteristics and soil texture for forest–meadow ecotones with different land uses (*n* = 9; bold and italics indicate a significant correlation at * *p* ≤ 0.05, ** *p* ≤ 0.01).

Parameter	Reserve	Pasture
Plant litter quantity and quality		
Grass AGB, kg C m^−2^	***0.86 *****	0.54
Total plant litter ^1^, kg C m^−2^	0.45	−0.18
Aromatic C, %	−0.35	−0.65
Aromaticity	−0.36	**−** ***0.68 ****
C/N ratio	***0.69 ****	0.55
Soil texture		
Silt, %	−0.58	-0.18
Clay, %	**−** ***0.79 *****	-0.60

^1^ Grass AGB + forest litter.

**Table A4:** Characteristics of grass cover per 0.5 × 0.5 m plot in mid-August 2020 (mean ± SE for *n* = 3) for forest–meadow ecotones with different land uses. Notes: PC, projective cover; NS, number of species.

Site	PC (%)	NS	Dominant Species (≥10% of PC)
Reserve			
Meadow	98 ± 2	12 ± 1	*Calamagrostis arundinacea*, *Stachys macrantha*, *Ranunculus caucasicus* subsp. *subleiocarpus*, *Astrantia maxima*
Tree line	62 ± 6	7 ± 1	*Calamagrostis arundinacea*, *Pulsatilla aurea*, *Veratrum album*, *Gentiana septemfida*
Forest	40 ± 6	8 ± 1	*Aconitum orientale*, *Prenantes abietina*, *Myosotis amoena*, *Petasites albus*
Pasture			
Meadow	93 ± 2	10 ± 0	*Nardus stricta*, *Cirsium obvalatum*, *Potentilla erecta*, *Agrostis capillaris*, *Stachys macrantha*
Tree line	75 ± 9	9 ± 2	*Agrostis capillaris*, *Prunella vulgaris*, *Myosotis amoena*
Forest	53 ± 7	10 ± 0	*Alchemilla vulgaris*, *Oxalis acetosella*, *Trifolium repens*
